# *Klebsiella pneumoniae* invasive syndrome with liver abscess and purulent meningitis presenting as acute hemiplegia: a case report

**DOI:** 10.1186/s12879-023-08383-w

**Published:** 2023-06-12

**Authors:** Yu Chang, Jiann-Hwa Chen, Wei-Lung Chen, Jui-Yuan Chung

**Affiliations:** 1 Department of Emergency Medicine, Cathay General Hospital, 280, Sec. 4, Ren’ai Rd., Da’an Dist., Taipei City 106, Taipei, Taiwan; 2 School of Medicine, Fu Jen Catholic University, Taipei, Taiwan; 3 School of Medicine, National Tsing Hua University, Hsinchu, Taiwan

**Keywords:** Klebsiella pneumoniae, Liver abscess, Meningitis, Hemiplegia

## Abstract

**Background:**

*Klebsiella pneumoniae* can infect a variety of sites, with the risk of infection being higher in the immunocompromised state such as diabetes mellitus. A distinct invasive syndrome has been detected mostly in Southeast Asia in the past two decades. A common destructive complication is pyogenic liver abscess that can be complicated by metastatic endophthalmitis as well as the involvement of the central nervous system, causing purulent meningitis or brain abscess.

**Case presentation:**

We report a rare case of an invasive liver abscess caused by K. pneumoniae, with metastatic infections of meninges. A 68-year-old man with type 2 diabetes mellitus presented to our emergency department as sepsis. Sudden disturbed consciousness was noticed with presentation of acute hemiplegia and gaze preference mimicking a cerebrovascular accident.

**Conclusions:**

The above case adds to the scarce literature on K. pneumoniae invasive syndrome with liver abscess and purulent meningitis. K. pneumoniae is a rare cause of meningitis and should raise suspicions about the disease in febrile individuals. In particular, Asian patients with diabetes presenting with sepsis and hemiplegia prompt a more thorough evaluation with aggressive treatment.

**Supplementary Information:**

The online version contains supplementary material available at 10.1186/s12879-023-08383-w.

## Background

*Klebsiella pneumoniae* can infect a variety of sites, with the risk of infection being higher in the immunocompromised state such as diabetes mellitus. Most community-acquired *K. pneumoniae* infections cause pneumonia and urinary tract infections. However, a distinct invasive syndrome has been detected mostly in Southeast Asia in the past two decades [1]. A common destructive complication is pyogenic liver abscess that can be complicated by metastatic endophthalmitis as well as the involvement of the central nervous system, causing purulent meningitis or brain abscess.

Pyogenic liver abscess has a global distribution, although incidence varies significantly between different Asian countries and non-Asian regions. The annual incidence of liver abscess has been estimated at 2.3 cases per 100,000 people in Canada and the United Kingdom [2, 3]; substantially higher rates have been reported in East Asian countries (up to 17.6 cases per 100,000) [4]. There is a wide range of pathogens associated with pyogenic liver abscesses, which can be attributed to various factors such as medical interventions and immunosuppression statuses, as well as regional differences. Both facultative and anaerobic species are commonly found. *Escherichia coli* and *K. pneumoniae* are frequently identified as pathogens [5], but a series of cases in the United States showed that the *Streptococcus milleri* group was the most commonly found pathogen [6, 7].

Liver abscesses in patients infected with *Klebsiella pneumoniae* were first described in the 1980s in anecdotal reports and case series from Taiwan [8]. A meta-analysis showed that the prevalence of *K. pneumoniae* infection has been increasing since the late 1980s and that it is now the main cause of liver abscess in Taiwan, accounting for almost 80% of cases [9]. Extrahepatic complications resulting from bacteremia, including endophthalmitis, meningitis, necrotizing fasciitis, and other illnesses, have also been recorded [10, 11]. Increased incidence of a distinct community-acquired invasive *K. pneumoniae* syndrome has been reported from Taiwan and other Southeast Asian countries [12–14]. However, this situation is still rare. A previous study found that only 1 (0.9%) out of 112 patients with K. pneumoniae liver abscess had central nervous system (CNS) involvement [15]. A rarer condition is *K. pneumoniae* meningitis presenting as acute hemiplegia. Prompt identification and treatment are necessary to reduce patient morbidity and prevent mortality.

We report a rare case of an invasive liver abscess caused by *K. pneumoniae*, with metastatic infections of meninges, presenting with acute hemiplegia and gaze preference mimicking a cerebrovascular accident.

### Case presentation

A 68-year-old man with type 2 diabetes mellitus and deep vein thrombosis, who had refused medical treatment for personal reasons for one year, presented at the emergency department after two weeks of general weakness. The patient had been in his usual state of health until the day before the current evaluation. He is a smoker but rarely drinks alcohol and does not use illicit drugs. On presentation, his initial vital signs included a body temperature of 39.4 °C, heart rate of 118 beats per minute, respiratory rate of 22 breaths per minute, blood pressure of 88/53 mmHg, and oxygen saturation of 96% on ambient air. On physical examination, he appeared to be normal waking, uncomfortable, and diaphoretic, with dry mucous membranes, warm extremities, and mild tenderness on palpation of his upper abdomen.

Septic shock was first suspected. The complete blood count demonstrated leukocytosis with a white blood cell count(WBC) of 12.95 × 103/uL, neutrophil predominance of 77.4% segments and 10.4% bands, and thrombocytopenia indicated by a platelet count of 26 × 103/uL without evidence of anemia. Laboratory analysis also revealed an alanine transaminase level of 103 IU/L, creatinine of 1.60 mg/dL, glucose of 496 mg/dL, C-reactive protein of 24.016 mg/dL, and lactic acid of 4.5 mmol /L. Venous blood gas results were as follows: pH of 7.363, PaCO2 of 26.4 mmHg, PaO2 of 30.8 mmHg, HCO3 of 14.7 mmol/L, and ketone of 5.6 mmol/L. Blood osmolality was 288 mOsm/kg. The coagulation profile showed a prothrombin time of 12.6 s, an international normalized ratio of 1.13, and an activated partial thromboplastin time of 31.3 s. Urinalysis showed 6–10 red blood cells and 6–10 white blood cells per high-powered field with positive leukocyte esterase and ketone body. Peripheral blood cultures were collected twice from separate sites. Suspecting septic shock and diabetic ketoacidosis, we prescribed broad-spectrum empiric antibiotics with Flomoxef sodium and continuous intravenous insulin infusion.

**Fig. 1:**
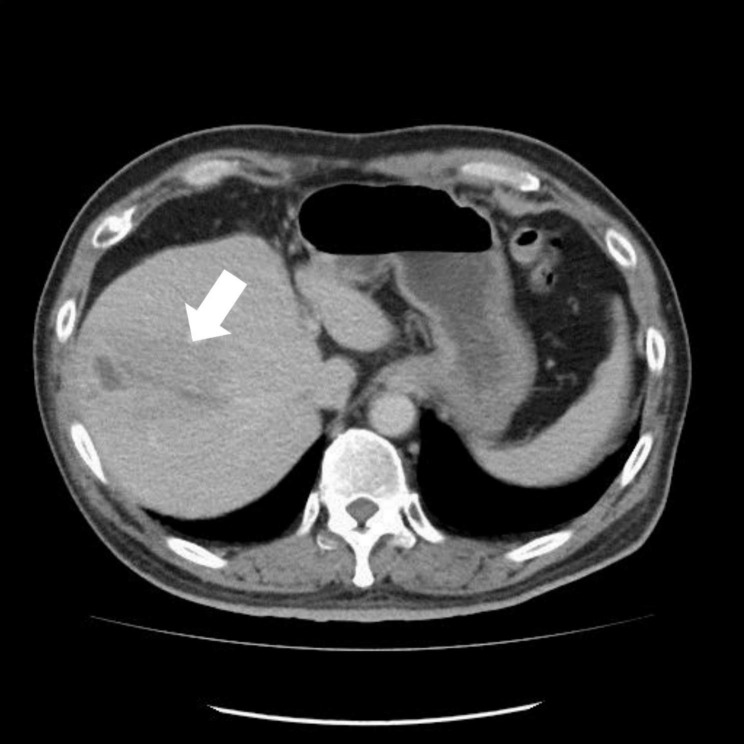
A lobulated hypodense and hypoenhancing hepatic mass (arrow) with internal necrotic areas located at S8 causing thrombosis of an intrahepatic portal vein

A contrast-enhanced abdominal computerized tomography (CT) scan showed a lobulated hypodense mass (93 × 53 × 48 mm) with hypoenhancement and some internal eccentric liquefied areas at S7, S8, and S5 of the liver, where thrombosis of the anterior superior branch of the intrahepatic portal vein was noted, which suggested a possible liver abscess (Fig. 1). After the patient received medical care for about six hours at our emergency department, sudden disturbed consciousness was noticed by the family with presentation of conjugate eye deviation to the left and severe left-sided hemiparesis (muscle strength grading of 1/5). The patient could withdraw his right arm and right leg in response to pain, and a jerking motion of the head was noted. The pupils were 2.5 mm, symmetrical, and reactive to light. The score on the Glasgow Coma Scale was 6 (E1M4V1, with lower scores indicating greater alteration of consciousness).

A differential diagnosis of septic cerebral emboli resulting in ischemic stroke was first considered. CT angiography of the cerebral arteries showed cerebrovascular atherosclerosis without obvious vessel occlusion or thrombosis. The patient underwent a lumbar puncture to rule out meningitis. The cerebrospinal fluid (CSF) appeared yellow and turbid. CSF analysis revealed a WBC count of 4141 cells/µL, with 87% neutrophils and 3% lymphocytes, protein level of 414 mg/dL, and glucose level of 59 mg/dL. Puncture examination showed that the opening pressure was 50 cm H2O. The lumbar puncture result was consistent with bacterial meningitis. CSF was submitted for Gram staining and bacterial culture. The patient was preliminarily diagnosed with a primary liver abscess and meningitis. Owing to his severely impaired consciousness and critical illness, he received endotracheal intubation and was admitted to the intensive care unit for further medical treatment. Antibiotic therapy was escalated to Doripenem hydrate 1000 mg intravenously every 12 h on the recommendation of the infectious disease specialist. Two sets of blood culture were reported as *K. pneumoniae*. The susceptibility test was examined which revealed sensitive to several penicillins, cephalosporins, aminoglycosides, quinolones, cotrimoxazole, and carbapenems (Supplementary Table 1). Urine cultures also revealed *K. pneumoniae*. Culture from CSF was negative. Nucleic acid amplification testing for herpes simplex virus type 1 and type 2 DNA was negative, and Gram staining revealed no organisms. No acid-fast bacilli were observed on a mycobacterial smear, and no fungi were seen on examination of a fungal wet preparation. Tests of CSF for *cryptococcal* antigen, *Streptococcus pneumoniae* antigen, and *meningococcal* antigen were negative.

**Fig. 2:**
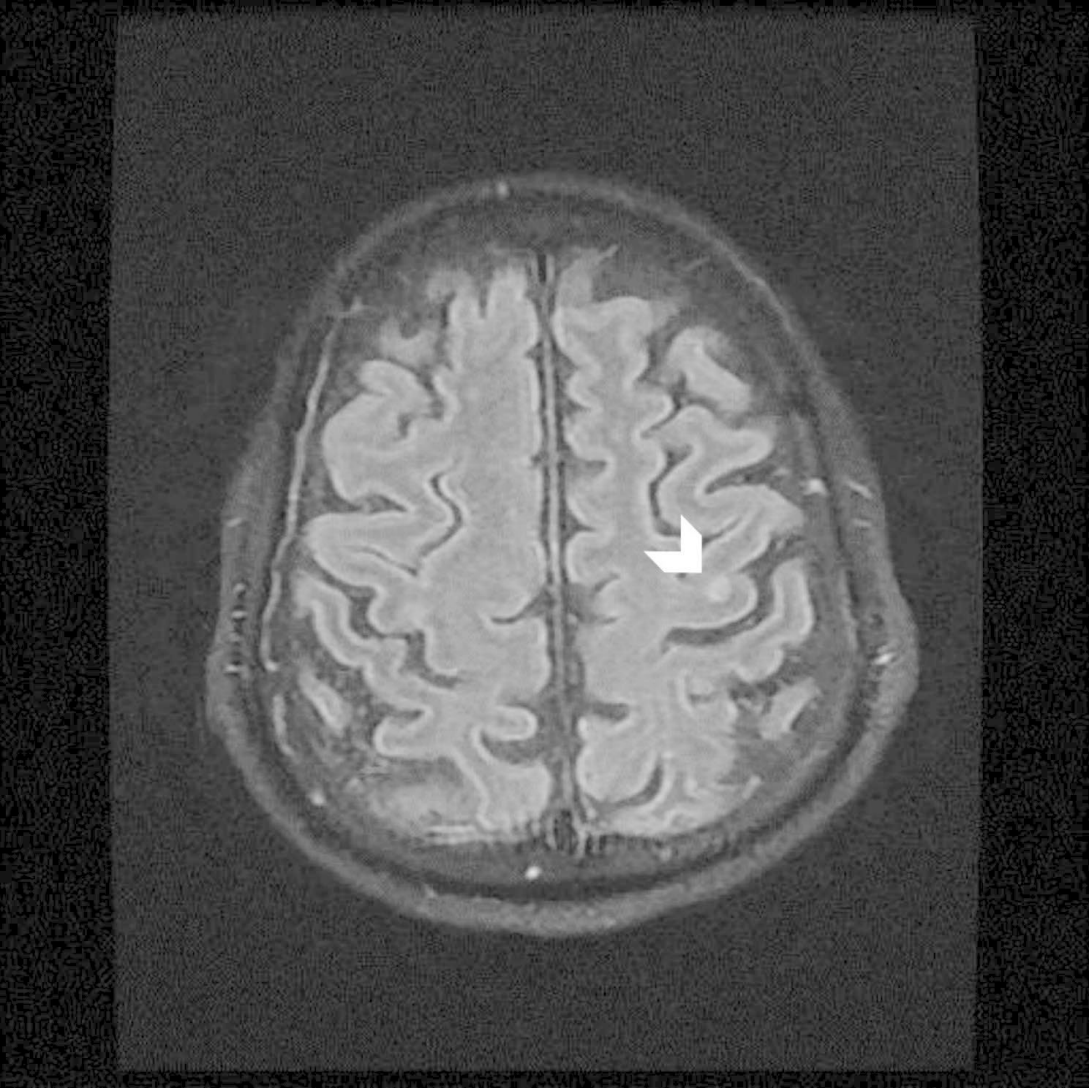
A 0.4 cm nodular enhancement at left higher frontal lobe (arrow head)

During admission, gastroenterologist was consulted, and the specialist did not recommend draining the abscess due to its immature and non-liquefied form of liver abscess. The patient’s condition gradually stabilized under intensive treatment. On the sixth hospital day, he fully recovered consciousness. He was successfully extubated and transferred to a general ward on the 10th day. A brain magnetic resonance imaging scan with contrast was performed after extubation. The report showed one 0.4-cm nodular enhancement in the left higher frontal lobe, without an increase in the diffusion-weighted image; microabscess was thus suspected (Fig. 2). Despite no visual complaint by the patient, we consulted an ophthalmologist to detect intraocular diseases and exclude endophthalmitis, which was confirmed by funduscopic examination. Transthoracic echocardiogram revealed normal contractility without vegetation. The patient unexpectedly became infected with SARS-CoV-2 virus on day 25. He was then transferred to a negative pressure isolation ward for further antibiotic treatment. After six weeks of antibiotic treatment, he was successfully discharged directly from the isolation ward. No focal neurological deficit was observed.

## Discussion and conclusion

Several studies have reported that the clinical presentation of *K. pneumoniae* invasive syndrome can vary widely, depending on the underlying infection site [12–14]. For instance, patients with *K. pneumoniae* liver abscess may present with fever, abdominal pain, and jaundice, while those with pneumonia may present with cough, dyspnea, and chest pain. Patients with sepsis or meningitis may present with fever, altered mental status, and other systemic signs of infection.

The definition of primary liver abscess can vary between studies. In one study cited, primary liver abscess was defined as a liver abscess caused by *Klebsiella pneumoniae* occurring in the absence of any predisposing factors such as hepatobiliary disease, colorectal disease, or a history of intraabdominal surgery or trauma [16]. However, other studies may have different criteria for defining primary liver abscess. For example, some studies may define it as a liver abscess caused specifically by a single organism, such as *K. pneumoniae*, while polymicrobial liver abscesses are typically associated with underlying hepatobiliary disease or intraabdominal infection [1].

The pathogenesis of *K. pneumoniae* invasive syndrome is controversial. The reasons for the predominance of this syndrome in Asian populations are unclear. In 2002, Ko and colleagues found that *K. pneumonia*e isolated from Asian patients with the invasive syndrome had distinct phenotypic and genotypic features [17]. Several virulence factors have been described for *K. pneumoniae*, including the presence of the capsular serotype, mucoviscosity-associated gene A (magA), regulator of mucoid phenotype A gene (rmpA), and aerobactin [18]. The *K. pneumoniae* genotype K1 is an emerging pathogen capable of causing catastrophic septic ocular or CNS complications [16]. Lee et al. found that the presence of the rmpA gene, an Acute Physiologic and Chronic Health Evaluation (APACHE) II score of ≥ 20, and the presence of septic shock were important predictors of septic metastatic lesions in *K. pneumoniae* infection [19]. Diabetes mellitus or impaired fasting glucose is the major observed risk factor for *K. pneumoniae* primary liver abscess; these conditions are not major risk factors for liver abscesses caused by organisms other than *Klebsiella* [1, 20].

The optimal treatment for *K. pneumoniae* invasive syndrome depends on the specific infection site and severity of the disease. Antibiotic therapy is the mainstay of treatment for most cases of *K. pneumoniae* invasive syndrome, and empirical broad-spectrum antibiotics should be initiated promptly in suspected cases. In addition, drainage of abscesses and other infected sites may be necessary to achieve clinical improvement [21]. Despite there is limited evidence on the efficacy of doripenem for treating meningitis, it is a broad-spectrum antibiotic that has demonstrated effectiveness against various bacterial infections [22], and has been approved for the treatment of complicated intra-abdominal infections [23]. The decision to use doripenem was made by an infectious disease specialist after considering the patient’s severe sepsis and disseminated infection condition.

The mortality rate can vary widely, depending on the severity of the infection and the presence of underlying comorbidities. Several studies have reported mortality rates ranging from 5 to 40%, with the highest rates observed in patients with sepsis or meningitis [1]. However, prompt diagnosis and appropriate treatment can improve outcomes in these patients, and several studies have reported good outcomes with early antibiotic therapy and drainage of abscesses [6, 9, 12, 15].

*Streptococcus pneumoniae* is the most common cause of community-acquired meningitis in adults, while other pathogens’ distribution depends on patient age, vaccination status, and regional epidemiological trends [24, 25]. *K. pneumoniae* is an extremely rare cause of bacterial meningitis. While fever, nuchal rigidity, and change in mental status are classic symptoms, only two-thirds of patients diagnosed with bacterial meningitis present with all three [26]. Focal neurological abnormalities, seizures, and cerebral abnormalities are common during the clinical course. Hearing loss and seizures are the most frequent sequelae, with neurocognitive dysfunction as a less life-threatening possibility [27–29].

*K. pneumoniae* meningitis in adults is infrequently reported from Europe and the United States, in contrast to Taiwan, where it is most often hospital-acquired and associated with prior neurosurgical procedures or instrumentation [30, 31]. However, of 115 cases of *K. pneumoniae* meningitis reported in Taiwan, 84% were community acquired, and 64% of the cases had concurrent *Klebsiella* bacteremia [17].

The diagnosis of meningitis is challenging but crucial for emergency physicians. Delays in diagnosis and treatment can increase morbidity and mortality, with mortality rates reaching 70% without treatment [32]. Prognosis can be improved by early instigation of both antibiotic and steroid treatments [33].

The presented case adds to the scarce literature on *K. pneumoniae* invasive syndrome with liver abscess and purulent meningitis. Early identification and intervention are vital to reduce morbidity and prevent mortality due to this condition. *K. pneumoniae* is a rare cause of meningitis and should raise suspicions about the disease in febrile individuals. In particular, Asian patients with diabetes presenting with sepsis and hemiplegia prompt a more thorough evaluation with aggressive treatment.

## Electronic supplementary material

Below is the link to the electronic supplementary material.

Supplementary Material 1

## Data Availability

The datasets used and/or analyzed during the current study are available from the corresponding author on reasonable request.

## Abbreviations

CNS: Central nervous system
WBC: White blood cell count
CT: Computerized tomography
CSF: Cerebrospinal fluid
