# Supplementary material for: Klebsiella pneumoniae invasive syndrome with liver abscess and purulent meningitis presenting as acute hemiplegia: a case report
Source: BMC Infect Dis. 2023 Jun 12;23:397. doi: 10.1186/s12879-023-08383-w (PMC10259354; doi:10.1186/s12879-023-08383-w)
Supplement: Supplementary file 1 — Supplementary Material 1 [file 12879_2023_8383_MOESM1_ESM.pdf]

**Supplement table 1. Susceptibility test of *K. pneumoniae* isolated from the blood cultures**

| Antibiotic | Susceptibility | MIC (μg/mL) |
|---|---|---|
| Amikacin | S | <=2 |
| Ceftazidime | S | <=1 |
| Ciprofloxacin | S | <=0.25 |
| Cefmetazole | S | <=1 |
| Cefotaxime | S | <=1 |
| Cefazolin | S or I | <=4 |
| Ertapenem | S | <=0.5 |
| Cefepime | S | <=1 |
| Gentamicin | S | <=1 |
| Imipenem | S | <=0.25 |
| Levofloxacin | S | <=0.12 |
| Meropenem | S | <=0.25 |
| Sulbactam/Ampicillin | S | 4 |
| Co-trimoxazole | S | <=20 |
| Piperacillin-tazobactam | S | <=4 |

* S = Susceptible; I = Intermediate; MIC = minimum inhibitory concentration
